# Risk factors for the development of cirrhosis within 1-year in non-cirrhotic patients with HBV- related acute-on-chronic liver failure

**DOI:** 10.3389/fmed.2026.1743728

**Published:** 2026-03-10

**Authors:** Wenling Wang, Xiaolin Wang, Yu Wu, Huaibin Zou, Manman Xu, Yu Chen

**Affiliations:** 1 Fourth Department of Liver Disease, Beijing Youan Hospital, Capital Medical University, Beijing, China; 2 Beijing Key Laboratory of Liver Regeneration and Artificial Liver Transformation Research, Beijing, China

**Keywords:** acute-on-chronic liver failure, cirrhosis, hepatitis B virus, prognosis, risk factors

## Abstract

**Aims and Background:**

This study aimed to provide a preliminary clarification of the predictive factors for the development of cirrhosis within 1-year cirrhosis in non-cirrhotic HBV-associated acute-on-chronic liver failure (HBV-ACLF) patients and develop a risk stratification algorithm.

**Methods:**

Non-cirrhotic HBV-ACLF patients who survived for at least 1 year and had complete clinical records were included from January 2016 to December 2023 at Beijing Youan Hospital, Capital Medical University. Multivariate logistic regression was performed to identify independent predictors of cirrhosis progression.

**Results:**

Of the 329 HBV-ACLF patients initially screened, 109 were enrolled in the study, and a 1-year follow-up revealed that 21.1% of the non-cirrhotic patients developed cirrhosis. Multivariate logistic regression identified independent predictors of cirrhosis progression: non-resolution of total bilirubin (TB) levels within 28 days [5.64 (1.39–25.00)], failure to normalize the international normalized ratio (INR) within 28 days [6.34 (1.52–29.50)], and baseline platelet (PLT) count [0.98 (0.97–0.99)]. ROC analysis demonstrated strong predictive accuracy for cirrhosis with INR normalization (AUC = 0.82), TB resolution (AUC = 0.78), and baseline PLT count (AUC = 0.75). A risk stratification pathway incorporating INR normalization and baseline PLT effectively categorized patients into low, medium, and high-risk groups, with corresponding cirrhosis incidence rates of 5.4%, 29.4%, and 77.8%, respectively.

**Conclusion:**

The findings underscore the importance of INR normalization, TB resolution, and baseline PLT count as independent predictors of cirrhosis and provide a useful framework for clinical decision-making and early intervention.

## Introduction

Acute-on-chronic liver failure (ACLF) refers to the acute decompensation of liver function that arises from underlying chronic hepatitis or cirrhosis (1). Hepatitis B virus (HBV) infection is a predominant cause of chronic liver disease (2), and HBV-related ACLF (HBV-ACLF) is the most prevalent form of liver failure in China, 3-month spontaneous survival rate was 31.4 % (3, 4). The advent of antiviral therapy has markedly enhanced the management of liver failure, resulting in improved patient prognosis (5). Patients with ACLF admitted in the Asia-Pacific region are predominantly non-cirrhotic or do not exhibit extrahepatic organ failure at the time of admission (1). However, the effects of long-term antiviral therapy on the outcomes of cirrhosis in non-cirrhotic HBV-ACLF patients remain unreported. Previous prognostic models and novel biomarkers, such as neutrophil-lymphocyte ratio (6–8), have predominantly focused on predicting the onset of ACLF (9) or forecasting the short-term survival rates and guiding treatment during the acute phase (10–13). However, prognostication during the recovery phase is equally important, yet the incidence and determinants of cirrhosis progression remain poorly defined. Therefore, this exploratory study seeks to preliminarily identify risk factors associated with 1-year cirrhosis development in non-cirrhotic HBV-ACLF patients and to propose a risk stratification algorithm that may inform early clinical interventions.

## Methods

### Study population

This retrospective study included patients with HBV-ACLF who were admitted to Beijing Youan Hospital, Capital Medical University, between January 2016 and December 2023. The inclusion criteria were as follows: (1) patients aged 18 years or older; (2) positive for Hepatitis B surface antigen (HBsAg) and/or HBV DNA for more than 6 months; (3) HBV-ACLF was diagnosed based on the Chinese Group on the Study of Severe Hepatitis B (COSSH) ACLF criteria (14) on the basis of non-cirrhotic chronic liver disease. Non-cirrhotic status was determined according to clinical practice guidelines for the diagnosis and management of cirrhosis, based on baseline clinical data and detailed medical history; (4) abdominal CT examination conducted during hospitalization; (5) follow-up for a minimum of 1 year (15). Patients meeting any of the following exclusion criteria were not included: (1) development of ACLF on the basis of cirrhosis; (2) diagnosis of hepatocellular carcinoma (HCC) or other malignancies; (3) coinfection with other hepatitis viruses (hepatitis A, hepatitis C, hepatitis E and hepatitis D); (4) autoimmune liver disease; (5) alcoholic liver disease, drug-induced hepatitis, hyperthyroidism, poisoning and other causes of liver failure; (6) unstable period of cerebral infarction or combined with other serious complications; (7) death or receipt of a LT. All study procedures complied with the ethical principles of the Declaration of Helsinki. This research protocol received approval from the Ethics Committee of Beijing Youan Hospital, Capital Medical University. Written informed consent was obtained from all patients for the use of their medical records.

### Data collection

Demographic data, including gender and age, along with laboratory test results such as blood routine, liver function, kidney function, electrolytes, coagulation indicators, HBV virological markers, and quantitative HBV DNA were systematically collected at baseline and 28-day. Collected antiviral therapy information and followed up on patients' adherence to antiviral treatment. Prognostic scores, including the Child-Turcotte-Pugh (CTP) (16), the model for end-stage liver disease (MELD) (11), model for end-stage liver disease-sodium (MELD-Na) (17), the Chronic Liver Failure-Consortium (CLIF-C) ACLF (13), and the COSSH-ACLF II scores (10), were calculated based on the data obtained at the time of admission. A comprehensive evaluation was performed on clinical, biochemical, and imaging data—encompassing ultrasound, elastography, CT, MRI—pertaining to cirrhosis and/or portal hypertension, as well as relevant endoscopic findings.

### Diagnosis criteria of cirrhosis

The diagnosis of cirrhosis is established based on one or more of the following criteria , using clinical, laboratory, and imaging findings obtained at the 1-year follow-up (18): (1) Histological confirmation of cirrhosis; (2) Presence of gastroesophageal varices or ectopic varices in the digestive tract, provided that noncirrhotic portal hypertension has been excluded; (3) Imaging evidence of cirrhosis or portal hypertension, such as splenomegaly or a portal vein diameter of ≥1.3 cm; (4) liver stiffness measurement (LSM) results that meet the diagnostic cutoff for cirrhosis of various etiologies; and (5) Fulfillment of two or more of the following criteria: (a) PLT < 100 × 10^9^/L, with no alternative explanations; (b) Albumin (Alb) < 35 g/L, excluding malnutrition or renal disease; (c) INR >1.3 or prolonged prothrombin time, following the cessation of thrombolytic or anticoagulant therapy for more than 7 days; and (d) Aspartate aminotransferase (AST)/platelet ratio index (APRI): an APRI score >2 in adults.

### Selection of predictive variables

Both static and dynamic variables were analyzed. The dynamic variables included changes in INR, TB, cholinesterase (ChE), and PLT over a period of 28 days from onset (19–22). Based on a review of the literature, the criteria for evaluation were as follows (18, 23): INR normalization was defined as an INR value of less than 1.5 within 28 days (± 3 days); TB resolution was considered to be a reduction of more than 50% from the peak value within 28 days (± 3 days); PLT recovery was defined as a PLT count exceeding 100 × 10^9^/L within 28 days (±3 days); and ChE normalization was defined as ChE levels greater than 4000 U/L within 28 days (± 3 days).

### Statistical methods

Statistical analysis was conducted using R software (version *R* × 4.4.2). Normally distributed continuous variables are presented as mean ± standard deviation ($\bar{x}\pm s$), while non-normally distributed continuous variables are reported as median (interquartile range). Group comparisons were performed using independent *t*-tests or rank sum tests. Categorical data are expressed as frequency (proportions or rates), with group comparisons assessed through chi-square tests. Least absolute shrinkage and selection operator (LASSO) regression, as well as univariate and multivariate logistic regression, were employed to identify risk factors for the development of cirrhosis in patients with HBV-ACLF within 1 year. ROC curves were generated, and the Area Under the Curve (AUC) was calculated. In this study, the mean decrease in Gini was employed as the core metric to assess the relative importance of predictors in the random forest model. This helps identify key factors that significantly influence patient prognosis. A *P*- value of < 0.05 was deemed statistically significant.

## Results

### Clinical characteristics of non-cirrhotic HBV-ACLF patients

Among 329 screened HBV-ACLF patients, 152 were non-cirrhotic, of whom 43 died or underwent liver transplantation (LT). Of these, 31 (72.1%) experienced the event within 28 days, 10 (23.2%) between 28 and 90 days, and 2 (4.7%) between 90 days and 1 year. A total of 109 non-cirrhotic HBV-ACLF patients were ultimately included in the study, all of whom survived for more than 1 year. The majority were male (*n* = 94, 86.2%), with a mean age of 42.4 ± 10.5 years. After 1 year of follow-up, 23 patients (21.1%) in the recovery phase developed cirrhosis. Detailed information regarding complications, laboratory test results, changes in dynamic indicators, and baseline prognostic scores for the entire cohort is provided in Supplementary Table S1.

At baseline, the liver cirrhosis group exhibited significantly lower levels of Alanine Aminotransferase (ALT), AST, Alb, Hemoglobin (HB), and PLT, alongside a higher INR when compared to the non-cirrhotic group, with all differences reaching statistical significance (*P* < 0.05). Analysis of dynamic indicator over a 28-day period revealed that the probabilities of INR normalization, TB resolution, and PLT recovery were significantly lower in the cirrhosis group than in the non-cirrhotic group (*P* < 0.05). However, no significant difference was noted in the rate of ChE normalization between the two groups within the 28-day timeframe. Furthermore, a comparison of baseline fibrosis and prognostic scores indicated that the cirrhosis group had higher CTP, MELD, and MELD-Na scores at baseline compared to the non-cirrhotic group (*P* < 0.05). No significant differences were observed in the COSSH-II ACLFs and CTP grades (*P* > 0.05) (Table 1).

**Table 1:** Comparison of clinical characteristics and scores between cirrhosis and non-cirrhosis groups.

**Variables**	**Non-cirrhosis group (*n* = 86)**	**Cirrhosis group (*n* = 23)**	***P* value**
Male (*n*, %)	72 (83.7)	22 (95.7)	0.186
Age (years)	42.6 ± 11.0	41.6 ± 8.85	0.663
**Complications (** ***n*** **, %)**			
Ascites	46 (53.5)	16 (69.6)	0.471
BI	60 (69.8)	16 (69.6)	1.000
GIH	1 (1.2)	1 (4.4)	0.379
HE grade			0.200
Grade 0	74 (86.0)	19 (82.6)	
Grade I	11 (12.8)	2 (8.7)	
Grade II	1 (1.2)	2 (8.7)	
**Prognostic scores**			
**CTP**	**10 (9–11)**	**11 (11–12)**	**0.008**
**CTP score grading (** ***n*** **, %)**			0.274
Class B	23 (26.7)	3 (13.0)	
Class C	63 (73.3)	20 (87.0)	
COSSH-ACLF II	8.3 ± 0.7	8.5 ± 0.8	0.385
**MELD-Na**	**21.4 (19.1–23.7)**	**23.2 (21.5–24.6)**	**0.029**
**MELD**	**20.9** **±** **3.9**	**23.1** **±3.4**	**0.011**
**Antiviral drugs (** ***n*** **, %)**			0.452
ETV	31 (45.0)	12 (60.0)	
TAF	23 (33.3)	4 (20.0)	
ETV+TAF	15 (21.7)	4 (20.0)	
**Laboratory data**			
HBV-DNA (log_10_ IU/ml) (*n*, %)			0.448
< 4.0	30 (34.9)	10 (43.5)	
≥4.0	56 (65.1)	13 (56.5)	
HBeAg (+) (*n*, %)	43 (53.8)	10 (43.5)	0.527
TB (μmol/L)	285 (190–371)	338 (200–408)	0.694
**ALT (U/L)**	**551 (188–**1,161**)**	**180 (137–365)**	**0.011**
**AST (U/L)**	**292 (133–522)**	**149 (105–323)**	**0.041**
ALP (U/L)	141 (117–185)	160 (134–179)	0.480
GGT (U/L)	116 (76–155)	81 (48–139)	0.080
**Alb (g/L)**	**32.4** **±** **4.8**	**29.7** **±4.7**	**0.023**
GLO (g/L)	34.0 ± 7.7	34.6 ± 7.4	0.759
ChE (U/L)	3,741 ± 1,484	3,256 ± 1,196	0.178
**INR**	**1.9 (1.7–2.2)**	**2.3 (2.0–2.8)**	**0.003**
Cr (μmol/L)	61 (53–70)	61 (53–70)	0.730
BUN (mmol/L)	4.0 ± 1.3	4.0 ± 1.2	0.959
Na (mmol/L)	138 ± 3	138 ± 2	0.522
K (mmol/L)	4.0 ± 0.5	3.8 ± 0.5	0.299
Glu (mmol/L)	4.8 (4.2–6.3)	5.2 (4.4–6.2)	0.574
TG (mmol/L)	1.5 (1.1–2.0)	1.3 (0.9–1.8)	0.205
Tch (mmol/L)	3.1 (2.7–3.7)	2.7 (2.1–3.4)	0.149
**HB (g/L)**	**135** **±** **19**	**122** **±22**	**0.014**
WBC ( × 10^9^/L)	7.22 (5.27–8.66)	5.61 (4.38–8.90)	0.179
NEU ( × 10^9^/L)	4.90 (3.36–6.79)	3.45 (2.25–5.84)	0.065
LYM ( × 10^9^/L)	1.39 (1.09–1.84)	1.70 (1.31–2.31)	0.253
**PLT ( × 10** ^**9**^ **/L)**	**127 (104–177)**	**93 (60–110)**	**< 0.001**
AFP (ng/ml)	138 (56–384)	92 (30–179)	0.097
CRP (μg/ml)	9.5 (5.0–12.7)	11.3 (9.7–16.0)	0.063
**Dynamic variables (** ***n*** **, %)**			
**28-day INR**			**< 0.001**
**< 1.5**	**70 (81.4)**	**4 (17.4)**	
**≥1.5**	**16 (18.6)**	**19 (82.6)**	
**28-day TB reduction**			**< 0.001**
**≥50%**	**70 (81.4)**	**6 (26.1)**	
**< 50%**	**16 (18.6)**	**17 (73.9)**	
**28-day PLT**			**0.001**
≥100(**×10**^**9**^/L)	**42 (59.2)**	**4 (17.4)**	
< 100(**×10**^**9**^/L)	**29 (40.8)**	**19 (82.6)**	
**28-day ChE**			0.138
≥4,000 (U/L)	18 (26.9)	2 (9.5)	
< 4,000 (U/L)	49 (73.1)	19 (90.5)	

The data are expressed as medians (IQR), mean ± (SD) or number of patients (%). Boldface indicates variables with statistically significant differences between the two groups (*p* < 0.05); BI, bacterial infection; GIH, gastrointestinal hemorrhage; HE, hepatic encephalopathy; CTP, Child-Turcotte-Pugh score; MELD, model for end-stage liver disease score- MELD-Na, MELD-sodium score; COSSH-ACLF II, Chinese Group on the Study of Severe Hepatitis B-II ACLF score; ETV, Entecavir; TAF, Tenofovir alafenamide; HBV, hepatitis B virus; TB, total bilirubin; ALT, alanine aminotransferase; AST, aspartate aminotransferase; ALP, alkaline phosphatase; GGT, gamma-glutamyl transferase; Alb, albumin; GLO, globulin; ChE, cholinesterase; INR, international normalized ratio; BUN, blood urea nitrogen; Cr, creatinine; Na, serum sodium; K, serum potassium; Glu, fasting blood glucose; TG, triglycerides; Tch, total cholesterol; HB, Hemoglobin; WBC, white blood cell count; NEU, neutrophil; LYM, lymphocyte; PLT, platelet count; AFP, alpha-fetoprotein; CRP, C-reactive protein; PCT, procalcitonin.

### Identification of risk factors for the development of cirrhosis in HBV-ACLF patients in the recovery phase within 1 year

**Figure 1:**
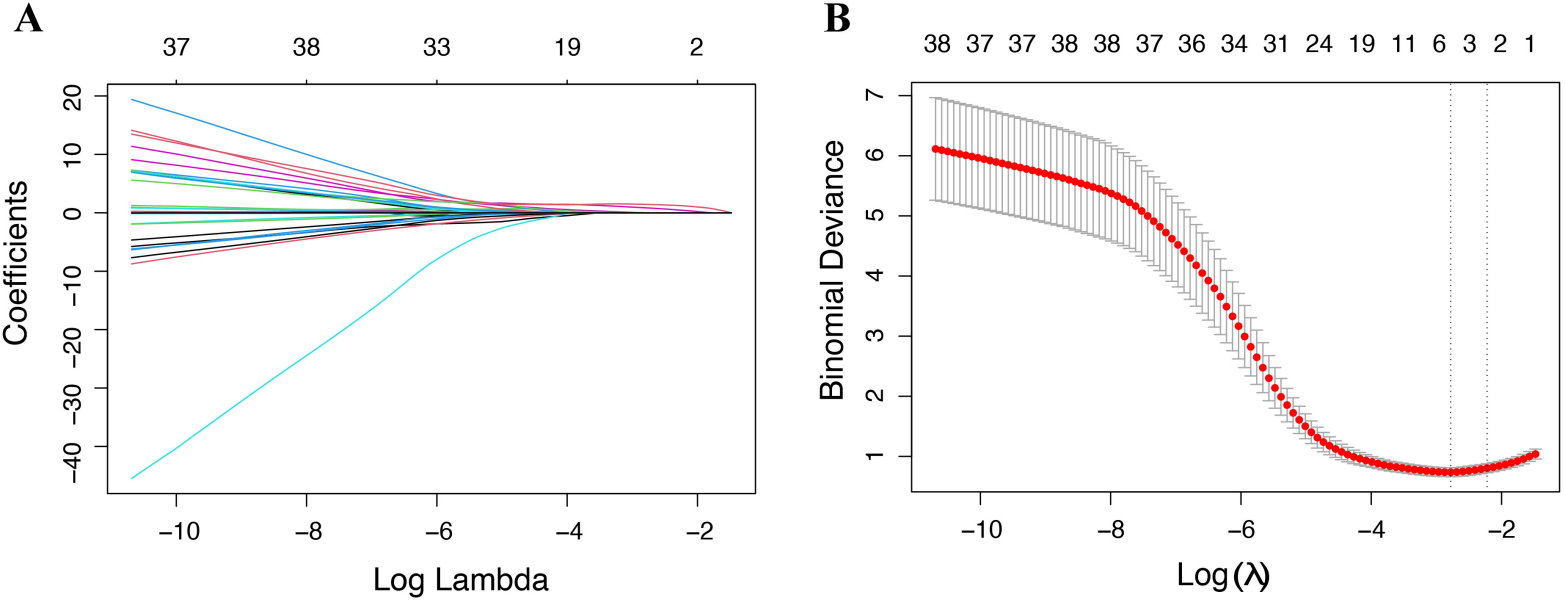
Coefficient paths for 1-year cirrhosis outcome in LASSO regression with varying Log(λ)**(A)**; Regularization path and risk factor selection based on LASSO regression **(B)**.

Clinical data from HBV-ACLF patients who developed cirrhosis were compared with those who did not. LASSO regression analysis identified four significant factors associated with this progression: INR normalization, TB resolution, PLT recovery, and baseline PLT count (Figure 1). The variables identified through LASSO regression were subsequently evaluated using both univariate and multivariate logistic regression analyses. The findings indicated that unresolved TB [5.64 (1.39–25.00)], failure to normalize the INR [6.34 (1.52–29.50)], and the baseline PLT [0.98 (0.97–0.99)] were independent risk factors for the development of cirrhosis in HBV-ACLF patients during the recovery phase (Table 2).

**Table 2:** Univariate and multivariate analysis of the association between LASSO-selected risk factors and 1-year liver cirrhosis.

**Variables**	**Univariate analysis**	**Multivariate analysis**
	**OR [95%CI]**	**OR [95%CI]**
**28-day TB reduction**		
**≥50%**	–	–
**< 50%**	**12.40 [4.43–39.20]**	**5.64 [1.39–25.00]**
**28-day INR**		
**< 1.5**	–	–
≥1.5	**20.78 [6.78–79.60]**	**6.34 [1.52–29.50]**
28-day PLT		
≥100( × 10^9^/L)	–	–
< 100( × 10^9^/L)	6.29 [2.15–23.11]	1.62 [0.34–8.26]
PLT(**×10**^**9**^/L)	**0.98 [0.97–0.99]**	**0.98 [0.97–0.99]**

INR, international normalized ratio; PLT, platelet count; TB, total bilirubin.

### Predictive accuracy of INR normalization, TB resolution, and baseline PLT for the development of cirrhosis within 1 year in HBV-ACLF patients without cirrhosis

**Figure 2:**
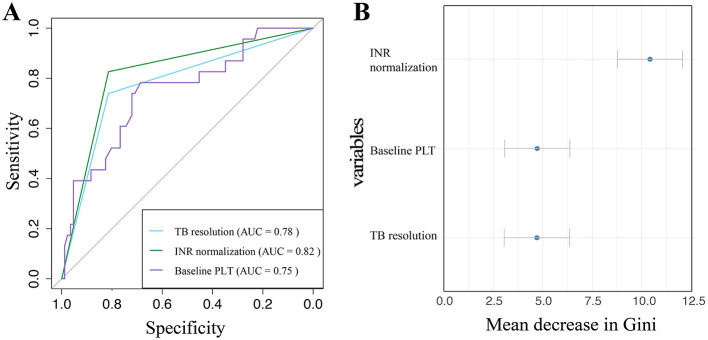
The ROC curve of INR normalization, TB resolution, and baseline PLT for predicting the development of cirrhosis within 1-year in non-cirrhotic HBV-ACLF patients **(A)**; Forest plot of the importance ranking of variables associated with the development of cirrhosis within 1-year **(B)**.

The results of the ROC analysis indicated that INR normalization exhibited an AUC of 0.82 (95% CI: 0.73–0.91) in predicting cirrhosis among ACLF patients without cirrhosis within 1-year. In comparison, the AUC for TB resolution was 0.78 (95% CI: 0.68–0.88), while the AUC for baseline PLT was 0.75 (95% CI: 0.63–0.87). These findings suggest that INR normalization has a more pronounced effect on the progression to liver cirrhosis and demonstrates superior predictive performance (Figure 2A).

### Risk prediction pathways for the development of cirrhosis within 1 year in HBV-ACLF patients without cirrhosis

The variables with *P* < 0.05 from both univariate and multivariate analyses were subjected to random forest regression analysis. The importance of each factor was ranked based on the mean Gini coefficient, with a higher index indicating better discrimination by the factor. The results indicated that the factors were ranked in the following order of importance: INR normalization, baseline PLT, and TB resolution (Figure 2B).

**Figure 3:**
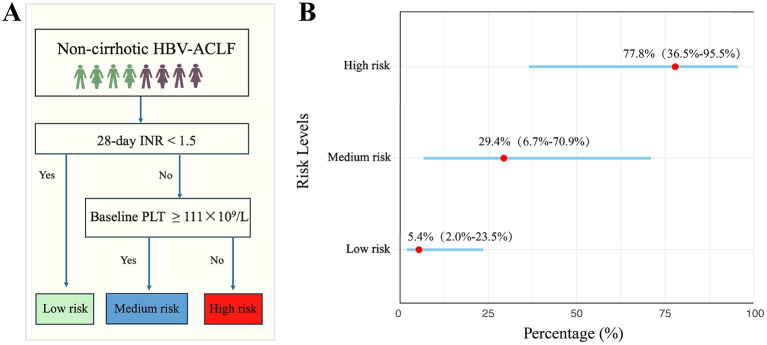
The pathway of 28-day INR changes in combination with baseline platelet count to stratify the risk of 1-year cirrhosis occurrence **(A)**. Gantt chart of different risk levels **(B)**.

The Sankey diagram shows that 74 of 109 patients (67.9%) had an INR < 1.5 at 28 days, of whom 4 (5.4%) developed cirrhosis within 1 year, while 70 (94.6%) did not. In contrast, among the 35 patients (32.1%) with an INR ≥1.5, 19 (54.3%) developed cirrhosis, and 16 (45.7%) did not (*P* < 0.001) (Supplementary Figure S1A). Regarding TB levels, 76 patients (69.7%) achieved TB resolution, with 6 (7.9%) developing cirrhosis within 1 year, and 70 (92.1%) remaining free of cirrhosis. In comparison, among the 33 patients (30.3%) who did not meet the criteria for TB resolution, 17 (51.5%) developed cirrhosis, and 16 (48.5%) did not (*P* < 0.001) (Supplementary Figure S1B). Concerning baseline PLT count, 67 patients (61.5%) had a PLT count ≥ 111, with 6 (9.0%) developing cirrhosis and 61 (91.0%) remaining cirrhosis-free. Among the 42 patients (38.5%) with PLT count < 111, 17 (40.5%) developed cirrhosis, and 25 (59.5%) did not (*P* < 0.001) (Supplementary Figure S1C). We established a risk stratification pathway by integrating INR normalization with baseline PLT count (Figure 3A). According to the clinical pathway, patients with an INR of less than 1.5 at 28 days were classified as low-risk group. In contrast, those who did not meet the INR < 1.5 criterion at 28 days and the baseline PLT count ≥111 were categorized into the moderate-risk group. Lastly, patients with an INR of less than 1.5 at 28 days and baseline PLT count of less than 111 were classified as high-risk group. The proportions of patients who developed cirrhosis within 1 year in the low, moderate, and high-risk groups were 5.4%, 29.4%, and 77.8% respectively. The probability of developing cirrhosis in the low-risk group was 5.4% (95% CI: 2.0%−23.5%), in the moderate-risk group was 29.4% (95% CI: 6.7%−70.9%), and in the high-risk group was 77.8% (95% CI: 36.5%−95.5%) (Figure 3B). Among the 12 patients who died or underwent liver transplantation between 28 days and 1-year, clinical pathway–based risk stratification showed that, if they had survived beyond 1 year, 1 patient would have been classified as low risk, 6 as intermediate risk, and 5 as high risk for cirrhosis development. These findings indicate that this subgroup remains at a substantially high risk of progression to cirrhosis, even in the absence of death.

## Discussion

The recovery process from acute-on-chronic liver failure (ACLF) is both intricate and prolonged. In patients with HBV-related ACLF who do not have cirrhosis, some recover completely following treatment, while others progress to cirrhosis despite therapeutic intervention. Prior research has demonstrated that the median time to cirrhosis development after recovery in CHB-ACLF patients is approximately 12.5 months. Our study preliminarily explores two findings that may guide clinical decision-making in this challenging clinical situation. First, failure to achieve INR normalization, unresolved TB within 28 days and the baseline PLT count have been identified as independent risk factors for cirrhosis development within 1 year in non-cirrhotic HBV-ACLF patients. Second, we propose a clinical pathway for risk stratification that incorporates INR normalization and baseline PLT count to help detect high-risk patients, thereby facilitating timely intervention.

Chronic HBV infection poses a significant public health challenge, particularly in China, where it is a leading cause of chronic liver disease (2, 3, 19). HBV-ACLF is characterized by the acute decompensation of liver function in patients with chronic HBV infection, resulting in elevated morbidity and mortality rates (24, 25). This study monitored 109 patients with non-cirrhotic HBV-ACLF, with the objective of exploring risk factors for the development of cirrhosis within 1-year period. The results indicated that 21.1% of the patients developed cirrhosis, highlighting the critical need for early identification of high-risk groups. A comparative analysis of outcomes between patients who developed cirrhosis and those who did not revealed that INR normalization, TB resolution, and baseline PLT were independent risk factors.

The INR is a sensitive marker of hepatic synthetic function (26, 27). Failure to normalize within 28 days indicates severe impairment of liver synthesis. Accordingly, INR is incorporated into prognostic models for ACLF (10, 11, 13, 28). Previous studies have identified INR as an independent predictor of 28- and 90-day mortality in patients with cirrhosis and advanced fibrosis, with thresholds of 1.5 and 1.7 proposed as critical cut-offs for disease progression (22). In this study, we further demonstrated the prognostic value of INR in predicting long-term progression to cirrhosis among patients with HBV-ACLF without prior cirrhosis. Normalization of INR reflects recovery of hepatic synthetic function and may indicate improved hepatocyte regeneration and attenuation of fibrosis progression (26). Moreover, coagulation dysfunction can be exacerbated by systemic inflammation and infection (29). Therefore, INR normalization by day 28 may signify better immune regulation and reduced hepatic inflammation, potentially slowing disease progression and lowering the risk of cirrhosis. Consistently, 28-day INR normalization showed strong predictive value for 1-year cirrhosis (AUC = 0.82), underscoring the importance of early recovery of coagulation function.

TB is incorporated into several prognostic models for ACLF (11, 13, 14). A significant decline in TB levels generally reflects recovery of hepatic metabolic and excretory function and improved hepatocyte regeneration capacity (30, 31). Previous studies have shown that patients with cirrhosis exhibit higher baseline bilirubin levels than non-cirrhotic individuals (32, 33), and bilirubin has been identified as an independent predictor of fibrosis progression (34). Notably, a cohort study of 274 patients with HBV-ACLF demonstrated that elevated TB levels were associated with a higher risk of cirrhosis development (15). These findings indicate that impaired bilirubin conjugation and excretion reflect ongoing hepatic injury and fibrosis progression (35). Consistently, our results showed that failure of TB to decrease by >50% from peak levels within 28 days independently predicted cirrhosis at 1 year in non-cirrhotic HBV-ACLF patients, highlighting the importance of dynamic TB monitoring for early risk stratification and personalized management.

Thrombocytopenia is a common feature in patients with cirrhosis and is primarily attributed to splenic sequestration, reduced thrombopoietin synthesis, and impaired platelet production secondary to hepatic dysfunction (36–39). Previous studies have shown that a lower baseline PLT count is associated with poorer prognosis in patients with ACLF (40, 41). Beyond its hemostatic role, PLT plays a critical part in liver regeneration and immune modulation. Emerging evidence indicates that PLT is enriched in growth factors that promote hepatocyte proliferation and tissue repair, suggesting a direct contribution to liver regeneration (42, 43). In addition, immune dysregulation—a hallmark of ACLF—can adversely affect PLT function and survival through an imbalance of pro- and anti-inflammatory responses (44, 45). Metabolic disturbances, particularly in lipid and fatty acid metabolism, are also prevalent in ACLF and may further impair hepatic protein synthesis, including factors involved in thrombopoiesis (46). Accordingly, an increase in PLT during recovery may reflect hepatic functional improvement and favorable prognosis, whereas persistently low PLT levels indicate ongoing hepatic injury (37). Collectively, PLT may serve as an early surrogate marker of fibrosis progression during the recovery phase.

Our study demonstrated a progressive decline in the prognostic impact of 28-day INR normalization, PLT count, and TB resolution on cirrhosis development. Based on these findings, we propose a novel stratified risk assessment approach integrating INR normalization and baseline PLT to categorize patients into low-, intermediate-, and high-risk groups, enabling risk-adapted follow-up strategies in clinical practice. Specifically, patients in the low-risk group (28-day INR < 1.5) exhibited a low cirrhosis incidence (5.4%) and may be followed with routine outpatient monitoring. The intermediate-risk group (28-day INR ≥1.5 and baseline PLT ≥111 × 10^9^/L) showed a moderate risk (29.4%) and would benefit from intensified laboratory surveillance and imaging assessment. Notably, the high-risk group (28-day INR ≥1.5 and baseline PLT < 111 × 10^9^/L) demonstrated a striking cirrhosis incidence of 77.8%, suggesting the need for closer follow-up, early antifibrotic interventions. This algorithm can be readily incorporated into post-discharge follow-up workflows, allowing early identification of high-risk patients and timely optimization of individualized management strategies.

### Limitations of the study

Despite these significant findings, several limitations warrant acknowledgment. First, the single-center design may restrict the generalizability of our results. However, it is important to note that this research center is a nationally renowned hospital specializing in liver disease, attracting patients from across the country. Second, the 1-year follow-up period may not adequately capture the entire progression of cirrhosis, as some patients may progress beyond this timeframe. Future studies with larger cohorts and extended follow-up periods across multiple centers are necessary to validate our findings.

## Conclusion

This exploratory study finds that INR normalization, TB resolution, and baseline PLT count are independent predictors of cirrhosis development within 1 year in non-cirrhotic HBV-ACLF patients. By combining INR normalization and baseline PLT, a risk-stratified clinical pathway has been created, providing clinicians with a practical tool for timely identification of high-risk patients. This helps facilitate more personalized treatment and interventions to slow down the progression of liver fibrosis and improve long-term patient outcomes.

## Data Availability

Publicly available datasets were analyzed in this study. This data can be found here: The data that support the findings of this study are available from the corresponding author upon reasonable request.
