# Supplementary material for: Risk factors for the development of cirrhosis within 1-year in non-cirrhotic patients with HBV- related acute-on-chronic liver failure
Source: Front Med (Lausanne). 2026 Mar 10;13:1743728. doi: 10.3389/fmed.2026.1743728 (PMC13010861; doi:10.3389/fmed.2026.1743728)
Supplement: Supplementary file 1 [file Data_Sheet_1.docx]

Risk factors for 1-year cirrhosis in non-cirrhotic HBV-related acute-on-chronic liver failure patients

Wenling Wang^1,2*^, Xiaolin Wang^1,2*^, Yu Wu^1,2^, Huaibin Zou^1,2^, Manman Xu^1,2^, Yu Chen^1,2^

^1^Fourth Department of Liver Disease, Beijing Youan Hospital, Capital Medical University, Beijing, China.

^2^ Beijing Key Laboratory of Liver Regeneration and Artificial Liver Transformation Research, Beijing, China.

*These authors contributed equally to this work.

Corresponding authors:

Manman Xu, Fourth Department of Liver Disease, Beijing Youan Hospital, Capital Medical University, Beijing Key Laboratory of Liver Regeneration and Artificial Liver Transformation Research, NO.8 Xitou Tiao Road Youwai Street, Beijing, 100069, China. Email: xmm1903@ccmu.edu.cn.

Yu Chen, Fourth Department of Liver Disease, Beijing Youan Hospital, Capital Medical University, Beijing Key Laboratory of Liver Regeneration and Artificial Liver Transformation Research, NO.8 Xitou Tiao Road Youwai Street, Beijing, 100069, China. Email: chybeyond1071@ccmu.edu.cn.

**TABLE S1. ALL non-cirrhotic HBV-ACLF patient’s characteristics.**

| **Characteristics** | **ALL non-cirrhotic**  **HBV-ACLF (n=109)** |
| --- | --- |
| 1-year cirrhosis (n,%) | 23 (21.1) |
| Male (n,%) | 94 (86.2) |
| Age (years) | 42.4 ± 10.5 |
| **Complications** (n,%) |  |
| Ascites | 61 (56.0) |
| BI | 76 (69.7) |
| GIH | 2 (1.8) |
| HE grade |  |
| Grade 0 | 93 (85.3) |
| Grade I | 13 (11.9) |
| Grade II | 3 (2.8) |
| **Baseline prognostic / fibrosis score** |  |
| CTP | 10 (10-12) |
| CTP score grading (n,%) |  |
| Class B | 26 (23.9) |
| Class C | 83 (76.1) |
| COSSH-II ACLF | 8.4 ± 0.7 |
| MELD-Na | 21.6 (19.3-24.0) |
| MELD | 21.3 ± 3.9 |
| **Antiviral drugs** (n,%) |  |
| ETV | 43 (48.3) |
| TAF | 27 (30.3) |
| ETV+TAF | 19 (21.3) |
| **Laboratory data** |  |
| HBV-DNA (log_10_ IU/ml) (n,%) |  |
| < 4.0 | 86 (78.9) |
| ≥ 4.0 | 23 (21.1) |
| HBeAg (+) (n,%) | 53 (51.5) |
| TB (μmol/L) | 288 (193-376) |
| ALT (U/L) | 429 (159-998) |
| AST (U/L) | 248 (118-506) |
| ALP (U/L) | 142 (117-180) |
| GGT (U/L) | 114 (73-151) |
| Alb (g/L) | 31.8 ± 4.9 |
| GLO (g/L) | 34.2 ± 7.6 |
| ChE (U/L) | 3645 ± 1438 |
| INR | 2.0 (1.7-2.4) |
| Cr (μmol/L) | 61 (53-70) |
| BUN (mmol/L) | 4.0 ± 1.3 |
| Na (mmol/L) | 138 ± 3 |
| K (mmol/L) | 3.9 ± 0.5 |
| Glu (mmol/L) | 4.9 (4.3-6.4) |
| TG (mmol/L) | 1.4 (1.0-1.9) |
| Tch (mmol/L) | 3.0 (2.6-3.7) |
| HB (g/L) | 132 ± 20 |
| WBC ($\times{10}^{9}$/L) | 6.92 (5.06-8.68) |
| NEU ($\times{10}^{9}$/L) | 4.69 (3.20-6.54) |
| LYM ($\times{10}^{9}$/L) | 1.42 (1.12-1.87) |
| PLT ($\times{10}^{9}$/L) | 118 (93-164) |
| AFP (ng/ml) | 133 (55-354) |
| CRP (μg/ml) | 10.1 (6.2-14.7) |
| **Dynamic variable** (n,%) |  |
| 28-day INR |  |
| < 1.5 | 74 (67.9) |
| ≥ 1.5 | 35 (32.1) |
| 28-day TB reduction |  |
| ≥ 50% | 76 (69.7) |
| < 50% | 33 (30.3) |
| 28-day PLT |  |
| ≥ 100（$\times{10}^{9}$/L） | 46 (48.9) |
| < 100（$\times{10}^{9}$/L） | 48 (51.1) |
| 28-day ChE |  |
| ≥ 4000（U/L） | 20 (22.7) |
| < 4000（U/L） | 68 (77.3) |

The data are expressed as medians (IQR), mean ± (SD) or number of patients (%).

AFP, alpha-fetoprotein; Alb, albumin; ALT, alanine aminotransferase; ALP, alkaline phosphatase; AST, aspartate aminotransferase; BI, bacterial infection; BUN, blood urea nitrogen; ChE, cholinesterase; COSSH-Ⅱ ACLF, Chinese Group on the Study of Severe Hepatitis B-Ⅱ ACLF score; Cr, creatinine; CRP, C-reactive protein; CTP, Child-Turcotte-Pugh score; ETV, entecavir; HB, Hemoglobin; HBV, hepatitis B virus; HE, hepatic encephalopathy; INR, international normalized ratio; GIH, gastrointestinal hemorrhage; GGT, gamma-glutamyl transferase; GLO, globulin; Glu, fasting blood glucose; K, serum potassium; LYM, lymphocyte; MELD, model for end-stage liver disease score; MELD-Na, MELD-sodium score; Na, serum sodium; NEU, neutrophil; PLT, platelet count; PCT, procalcitonin; TAF, tenofovir alafenamide; TB, total bilirubin; Tch, total cholesterol; TG, triglycerides; WBC, white blood cell count.

**
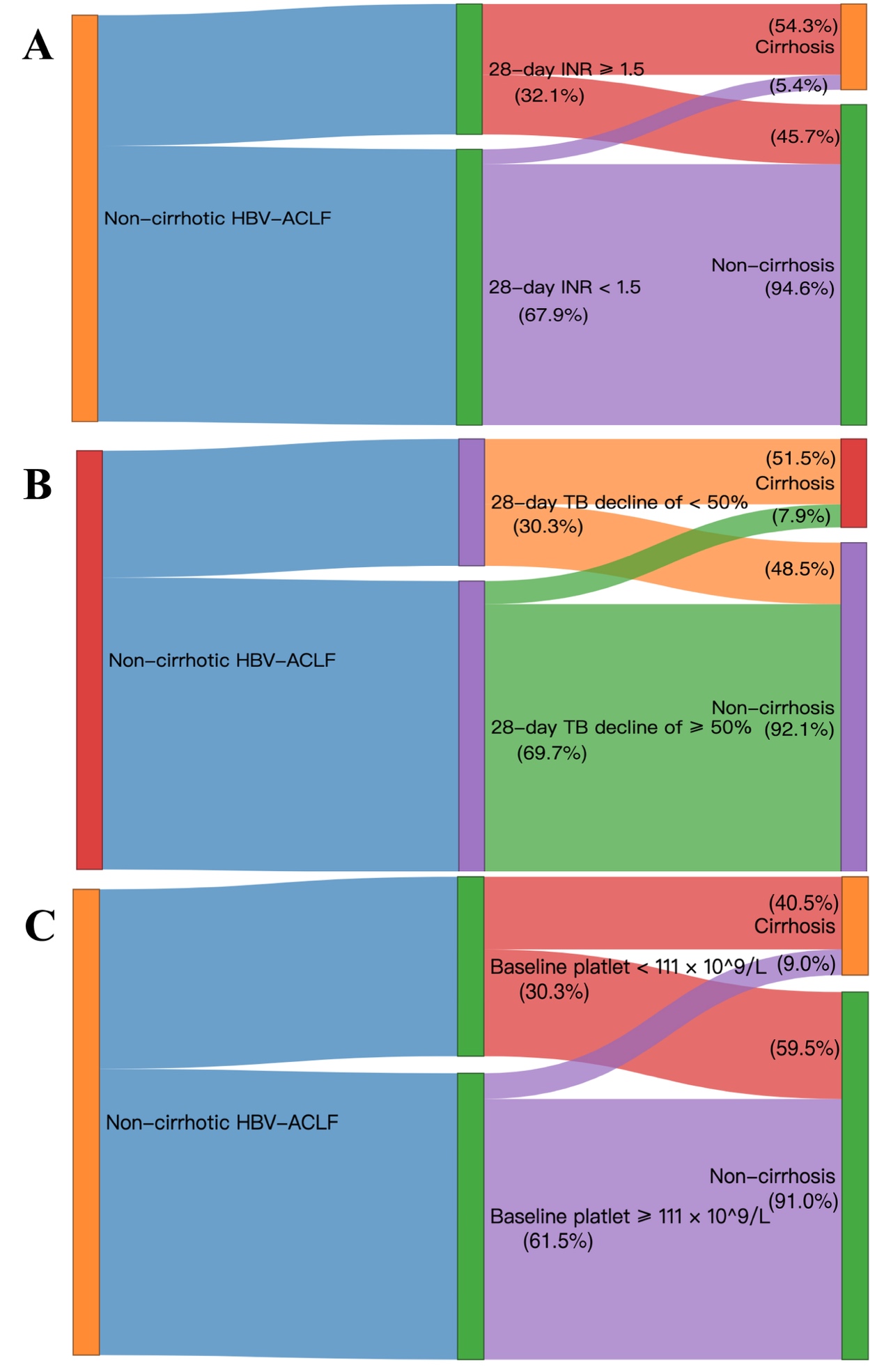
FIGURE S1.** **Sankey diagram analysis of 28-day changes in INR, TB, and baseline PLT count for predicting 1-year cirrhosis outcomes in non-cirrhotic HBV-ACLF patients.** HBV, hepatitis B virus; ACLF, acute-on-chronic liver failure; INR, international normalized ratio; TB, total bilirubin.
